# Temporal context effects on suboptimal choice

**DOI:** 10.3758/s13423-024-02519-y

**Published:** 2024-05-17

**Authors:** Margaret A. McDevitt, Jeffrey M. Pisklak, Roger M. Dunn, Marcia L. Spetch

**Affiliations:** 1 McDaniel College, Westminster, MD 21157 USA; 2 University of Alberta, Edmonton, Canada; 3 San Diego State University, San Diego, CA USA

**Keywords:** Suboptimal choice, Temporal context, Initial-link duration, Conditioned reinforcement, SiGN model, Pigeon

## Abstract

Choice can be driven both by rewards and stimuli that signal those rewards. Under certain conditions, pigeons will prefer options that lead to less probable reward when the reward is signaled. A recently quantified model, the Signal for Good News (SiGN) model, assumes that in the context of uncertainty, signals for a reduced delay to reward reinforce choice. The SiGN model provides an excellent fit to previous results from pigeons and the current studies are the first to test a priori quantitative predictions. Pigeons chose between a suboptimal alternative that led to signaled 20% food and an optimal alternative that led to 50% food. The duration of the choice period was manipulated across conditions in two experiments. Pigeons strongly preferred the suboptimal alternative at the shorter durations and strongly preferred the optimal alternative at the longer durations. The results from both experiments fit well with predictions from the SiGN model and show that altering the duration of the choice period has a dramatic effect in that it changes which of the two options pigeons prefer. More generally, these results suggest that the relative value of options is not fixed, but instead depends on the temporal context.

**Supplementary Information:**

The online version contains supplementary material available at 10.3758/s13423-024-02519-y.

Imagine making repeated choices between two options that provide delayed reward with the same probability. Choice of one option leads to information about whether the reward is coming, but choice of the other option does not. You are more likely to prefer the informative option, and preference for information is also seen in other species (e.g., Bode et al., 2023; Hursh & Fantino, 1974). We also know that, all else being equal, humans and other animals prefer options that give higher probabilities of reward (e.g., Herrnstein & Loveland, 1975; Shanks et al., 2002). Predictions become less clear, however, when the option providing information is the one that provides less probable reward. In this case, choosing the informative option lowers the obtained reward, a behavior that has been variously called suboptimal choice (e.g., Spetch et al., 1990), paradoxical choice (e.g., Ajuwon et al., 2023), or costly curiosity (Rodriguez Cabrero et al., 2019).

This “suboptimal” choice has now been extensively studied in animals (see Dunn et al., 2024; McDevitt et al., 2016; Vasconcelos et al., 2018; Zentall, 2016, for reviews). Some animals have been shown to seek information even when that choice results in considerably less food (Dunn & Spetch, 1990; Hinnenkamp et al., 2017; Kendall, 1974; Spetch et al., 1994). For example, pigeons and starlings show extreme suboptimal preference in a choice between 20% and 50% reinforcement (e.g., McDevitt et al., 2022; Stagner & Zentall, 2010; Vasconcelos et al., 2015). In that procedure and in the present work (Fig. 1), outcomes are signaled on the 20% food alternative and not signaled on the 50% food alternative. Thus, completion of a choice schedule on the suboptimal (20%) alternative immediately leads to an informative terminal-link stimulus during the delay. That is, one stimulus is presented on trials ending with food and a different stimulus is presented on trials ending without food. Completion of a choice schedule on the optimal (50%) alternative also leads to immediate presentation of one of two stimuli, but they are not correlated with the food and no food outcomes. Overall, the suboptimal option provides information but less frequent food, while the optimal option provides no information but more frequent food.

**Fig. 1:**
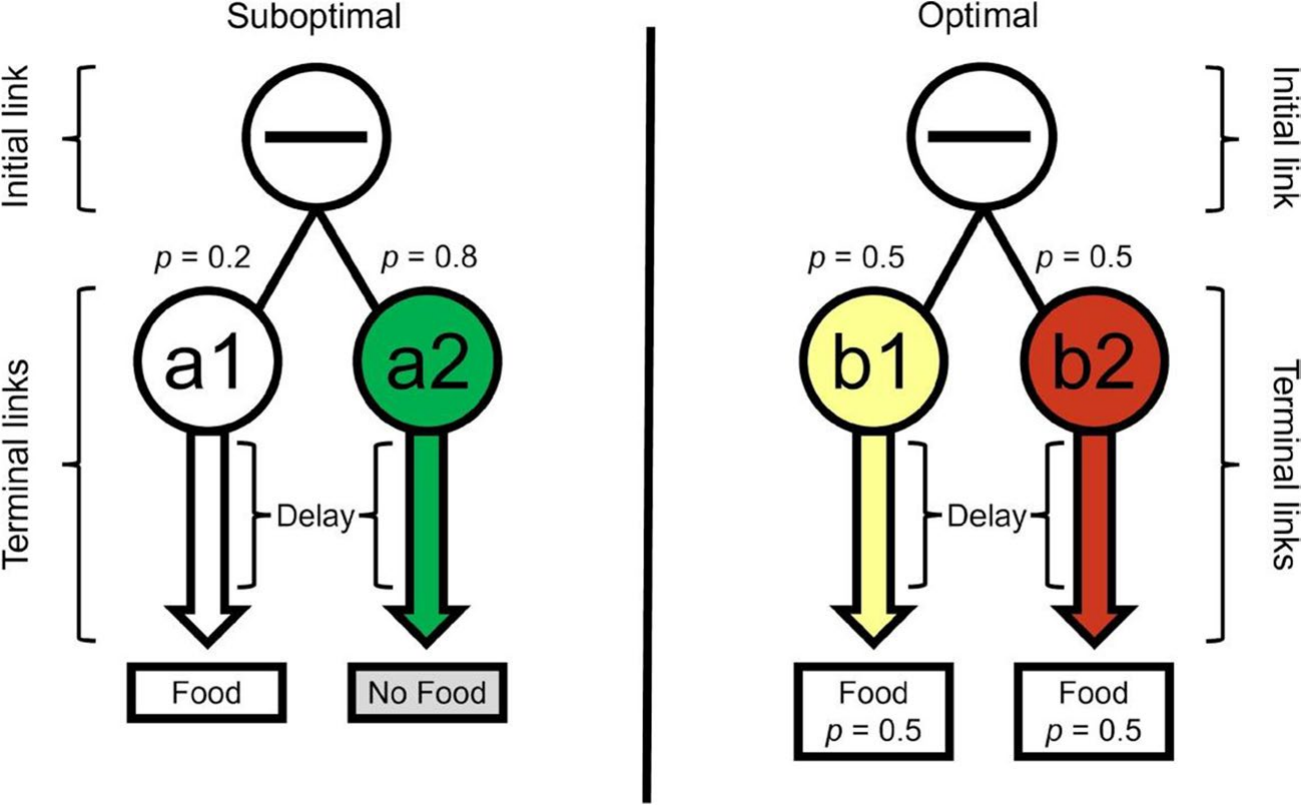
Experiment 1 procedure. *Note.* The suboptimal alternative was presented on one side key and the optimal alternative on the other, with side assignments counterbalanced across subjects. The suboptimal alternative led to one stimulus on 20% of the trials, which was always followed by food. Another stimulus was presented on the other 80% of the trials, which was always followed by blackout (no food). The optimal alternative led to one of two equally probable stimuli. Regardless of which stimulus was presented, the optimal alternative ended with food and blackout equally often. (Color figure online)

Recently, suboptimal choice has been the focus of renewed interest and a growing variety of theoretical perspectives (e.g., Ajuwon et al., 2023; Anselme, 2022, 2023; Daniels & Sanabria, 2018; González et al., 2020; Iigaya et al., 2016; McDevitt et al., 2016; Orduña & Alba, 2020; Vasconcelos et al., 2015; Zentall, 2016). Moreover, the tendency to choose options that provide signals for reward (noninstrumental information seeking) is increasingly of interest in neuroscience, cognitive science, and reinforcement learning (Blanchard et al., 2015; Bromberg-Martin & Monosov, 2020; FitzGibbon et al., 2020; Liew, Embrey, & Newell, 2023b; Rodriguez Cabrero et al., 2019).

One approach to understanding suboptimal choice, the Signal for Good News (SiGN) model (McDevitt et al., 2016), is an extension of the Delay Reduction Hypothesis (Squires & Fantino, 1971), which posits that stimuli that signal a reduction in waiting time to food reward become conditioned reinforcers. This hypothesis successfully describes choice between different delays to food but was not specified to account for choice in probabilistic procedures (i.e., when there is uncertainty about *whether* food will occur). Dunn and Spetch (1990) postulated that when outcomes are delayed and uncertain, signals for food provide extra conditioned reinforcement. Dunn et al. (2024) quantified the SiGN model and found that it provided an excellent fit to the results of 33 existing publications (128 data points) on pigeons and starlings. A unique feature of this model is that it has no free parameters and therefore quantitative predictions can be generated based on procedural variables alone. The current experiments provide the first test of quantitative a priori predictions.

In the SiGN model, temporal parameters are critical because stimuli signaling a reduction in delay to reward function as conditioned reinforcers. Other things being equal, longer delays to food (terminal-link schedules shown in Fig. 1) are more likely to generate preference for the suboptimal alternative in pigeons (McDevitt et al., 2018; Spetch et al., 1990, 1994). Moreover, preference for information about rewards also increases as a function of delay to reward in other species, including humans (e.g., Cunningham & Shahan, 2018; Iigaya et al., 2016, 2020; Liew, Embrey, Navarro, et al., 2023a, and see review by Dunn et al., 2024).

Although there is some suggestion in the literature that the duration of the choice phase (initial link) may influence suboptimal choice, this evidence, as noted by Cunningham and Shahan (2018, 2020), is quite limited. Dunn and Spetch (1990) found that pigeons made fewer suboptimal choices when initial links were longer than a single peck, but preference varied considerably between birds. Pisklak et al. (2019) found less suboptimal choice when 25 pecks were required than when a single peck was required, which is suggestive of temporal effects because 25 pecks take longer to complete. In one of three conditions tested, Zentall et al. (2017) found more suboptimal choice with a 1-s than 20-s choice phase. However, their study was designed to investigate precommitment and included an additional link prior to the choice that differed between the two groups. Thus, the specific effect of the choice duration could not be independently evaluated. In sum, there are indications from the literature that initial-link duration is important, but conclusive evidence for an effect on suboptimal choice is lacking. Our model makes the a priori prediction that manipulating the duration of the choice period should not only alter suboptimal choice but should produce a complete reversal of pigeons’ preferences between two options.

The present experiments are unique in that they systematically assess how initial-link duration influences suboptimal choice and directly test novel quantitative predictions of the SiGN model. Demonstrating temporal context effects on suboptimal choice would provide an important connection to other examples of lawful relationships between temporal factors and behavior. For example, in operant conditioning, time to reinforcement is a critical determinant of reinforcer effectiveness with longer delays producing less effective reinforcement (e.g., Chung & Herrnstein, 1967; Mazur, 1988, 2000). Preference for a signaled reinforcement schedule over an equivalent unsignaled schedule has been found to decline with initial-link duration (Alsop & Davison, 1986; Hursh & Fantino, 1974). However, in those procedures, there was no cost associated with preference for an informative alternative. Temporal relationships are also important in Pavlovian (e.g., Gallistel & Gibbon, 2000; Miller & Barnet, 1993) and reinforcement learning (e.g., Ludvig et al., 2012).

Despite evidence that temporal factors are important for many behaviors, few models of suboptimal choice currently include temporal parameters, and the SiGN model is the only one to make a priori quantitative predictions about the effect of temporal variables. For example, the contrast explanation of suboptimal choice (Stagner & Zentall, 2010) focuses on differences in probability which provides no obvious predicted effect of temporal factors. The ∆*−*Σ hypothesis (González et al., 2020) is also based on probabilities with no provision for temporal parameters, although it was recently revised to account for some temporal effects (Macías et al., 2024). Firmly establishing temporal effects in suboptimal choice would indicate that the inclusion of temporal variables is a necessary feature of a successful model of suboptimal choice. Cunningham and Shahan (2018, 2020) attribute the absence of temporal factors in some models (e.g., Daniels & Sanabria, 2018; Zentall, 2016) to the limited and inconsistent evidence of initial-link effects and explicitly note that the literature would benefit from additional studies exploring the effects of initial-link duration.

The present experiments manipulate initial-link schedules to assess the effect of temporal context on pigeons’ suboptimal choice. The studies provide a direct test of the SiGN model prediction that increasing the duration of the choice phase reduces preference for the suboptimal alternative. Example calculations are in Appendix A, code for the SiGN model is on the Open Science Framework (Dunn et al., 2022), and an online calculator to generate predictions can be found online (https://jpisklak.shinyapps.io/SiGN_Calc/). The SiGN model predicts that the manipulation of choice duration will produce a complete reversal of preference.

## Experiment 1

The initial-link schedule was a fixed ratio (FR) 1 in one condition and a variable interval (VI) 30-s in a comparison condition. The SiGN model predicts exclusive preference for the suboptimal alternative with an FR 1 schedule and strong preference for the optimal alternative with a VI 30-s schedule.

### Method

#### Subjects

The subjects were ten adult pigeons with experience in concurrent chains and simple discrimination procedures and were cared for in accordance with the Guide for the Care and Use of Laboratory Animals (National Research Council, 2011). They were maintained at approximately 85% of their free-feeding weights by grain obtained during experimental sessions and immediate postsession feedings when necessary. The pigeons were housed in individual cages under a 12-hr light/dark cycle, with water and grit freely available. Bird 822 became ill and was removed from the experiment approximately halfway through the second condition, so data analyses include only the first condition for that bird.

#### Apparatus

Two operant chambers (approximately 360 mm wide, 320 mm long, and 350 mm high) were used. Three circular translucent response keys, 25 mm in diameter, were mounted on the front panel 260 mm above the floor and 72.5 mm apart. The center key was never used in these experiments. Each side key could be illuminated from the rear by standard IEE 28-V 12-stimulus projectors. A 28-V 1-W miniature lamp, located 87.5 mm above the center response key, provided general chamber illumination for the duration of each session, except during blackout periods as noted below. Directly below the center key and 95 mm above the floor was an opening (57 mm high by 50 mm wide) that provided access to a solenoid-operated grain hopper filled with mixed grain. When activated, the food hopper was raised for 5 s and illuminated from above with white light by a 28-V 1-W miniature lamp. A computer and a MED-PC interface, located in an adjacent room, controlled experimental events.

#### Procedure

##### Pretraining

Prior to beginning the experiment, each bird received pretraining for two to three sessions during which key pecks to the stimuli used in the experiment were reinforced according to a fixed-ratio (FR) schedule. To ensure that each subject was reliably pecking all stimuli before starting the experiment, the schedule was gradually increased from FR 1 to FR 20.

##### Training

An overview of the procedure is shown in Fig. 1. The suboptimal alternative was presented on one side key and consisted of a black horizontal line stimulus that, when chosen, was replaced with a color terminal-link stimulus (e.g., green or white keylight) that remained illuminated for 20 s. One terminal-link stimulus (e.g., white) was presented with a probability of .2 and was followed by a 5-s access to the food hopper. The other stimulus (e.g., green) was presented with a probability of .8 and was followed by 5-s termination of the houselight (blackout). Overall, the suboptimal alternative ended with food 20% of the time and the color of the terminal-link stimuli signaled which outcome would occur.

The optimal alternative was presented on the other side key and consisted of a black horizontal line stimulus that, when chosen, was replaced with a color terminal-link stimulus (e.g., yellow or red keylight) that remained illuminated for 20 s. Both terminal-link stimuli appeared equally often (*p* = *.*5) and half the time were followed by 5-s access to the food hopper and half the time by blackout. Thus, the optimal alternative ended with food 50% of the time, and the terminal-link stimuli did not differentially signal the outcomes.

The stimulus locations were constant (green and white on the left response key, yellow and red on the right), but the side associated with each alternative was counterbalanced across subjects, so that the optimal alternative was presented on the left for half of the birds and the right for the others. A 5-s intertrial interval separated each trial. Each session consisted of a combination of forced-exposure and choice trials. A forced-exposure trial consisted of the presentation of a single alternative (i.e., the initial-link stimulus on either the right or the left response key). Each block of three trials consisted of two forced-exposure trials (one suboptimal alternative, one optimal alternative) and one choice trial. The order of the trial types was randomized for each block, and sessions ended after a total of 30 trials or after 40 min, whichever occurred first.

##### Groups and initial-link schedules

The birds were randomly separated into two groups of five birds each. The initial-link schedule differed for the two groups. For one group, completion of an FR 1 schedule was required to enter a terminal link. That is, a single peck to an initial-link stimulus replaced it with a terminal-link stimulus as described above. For birds in the other group, completion of a VI 30 schedule was required to enter a terminal link. The VI operated using a single timer as in McDevitt and Williams (2001) and Roper and Zentall (1999). Thus, for birds in the VI 30 condition, the first peck following completion of the single timer determined which alternative was chosen. This use of a single timer, as opposed to the more common concurrent VI timers, is preferable because it removes the incentive to switch between alternatives (also known as changeover responses) that occurs with concurrent timers (see Pierce & Cheney, 2017, p. 295, for a description of this effect). Frequent switching behavior skews preference toward indifference and requires the addition of a changeover delay as a corrective measure. A single timer eliminates the need for a changeover delay. In addition, the use of a single timer reduces the discrepancy between the programmed and obtained schedule values that can occur with extreme preference levels and potentially bias preference. Thus, the single timer was used to eliminate these unintended influences on responding that might have complicated the interpretation of changes in preference as a function of changes in initial-link duration.

After 16 sessions (Phase 1), the horizontal line stimuli were replaced with squares, the initial-link schedules were switched for the two groups, and training continued for an additional 30 sessions (Phase 2).

#### Statistical analyses

For both Experiments 1 and 2, graphical and statistical analyses were conducted with R software (Version 4.3.2) using the *tidyverse* (Version 2.0.0), *RColorBrewer* (Version 1.1.3), *nlme* (Version 3.1.163), *rsq* (Version 2.6), and *effsize* (v0.8.1) packages (Neuwirth, 2022; Pinheiro & Bates, 2000; R Core Team, 2023; Torchiano, 2020; Wickham et al., 2019; Zhang, 2023). Data and R code are publicly available on the Open-Science Framework (McDevitt et al., 2023).

Within- (schedule) and between-subject (order) effects were analyzed using multilevel linear modeling fit with maximum likelihood estimation. Inverse Bayes factors (*BF*_10_) are provided to show how much support each main effect and interaction adds to the model. Differences in model *r*-squared values (*∆R*^2^_*M*_) are similarly provided to convey the importance of the included terms.

### Results

**Fig. 2:**
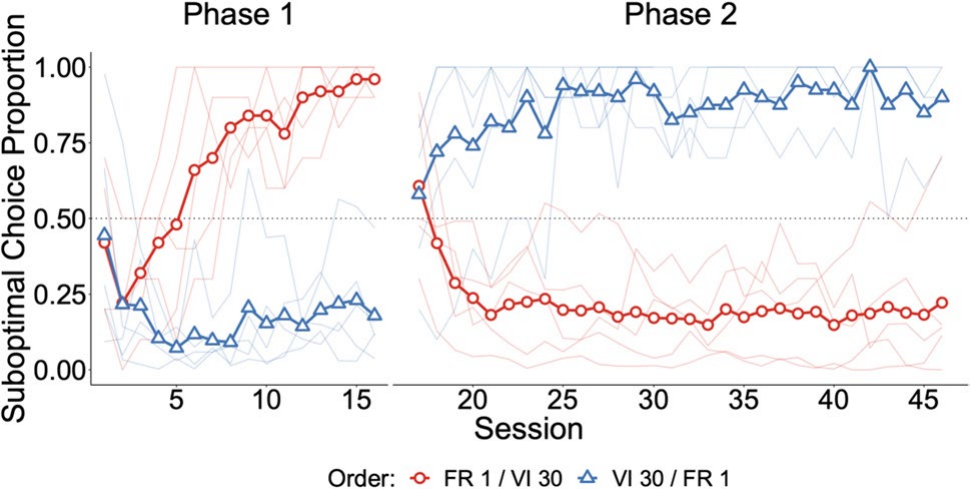
Experiment 1 results. *Note.* Suboptimal choice proportion across sessions. Bold lines represent mean choice proportion and thin lines represent individual subject data. Schedule order is denoted by both shape and color. FR 1 indicates a fixed-ratio 1 schedule and VI 30 indicates a variable-interval 30-s schedule in the initial links. (Color figure online)

Choice proportions for the suboptimal alternative were calculated using the last three sessions of each condition. Figure 2 shows the development of preference. Overall, the pigeons showed strong preference for the suboptimal alternative (*M* = *.*92, *SD* = *.*10) with an FR 1 initial link and strong preference for the optimal alternative with a VI 30-s schedule (*M* = *.*20, *SD* = *.*20). There was a significant main effect of schedule on suboptimal choice, *χ*^2^(1) = 35.95, *p* < .001, *BF*_10_ > 150, *∆R*^2^_*M*_ = .85. There was no main effect of order, *χ*^2^(1) = 0.08, *p* = .778, *BF*_10_ = 0.24, *∆R*^2^_*M*_ = .001; and no interaction, *χ*^2^(1) = 0.23, *p* = .630, *BF*_10_ = 0.26, *∆R*^2^_*M*_ = .002.

The alternative chosen on the first peck was recorded for birds with the VI initial link at the end of the second phase. The mean choice proportion (all pecks) for those subjects was .20 (*SD* = *.*23) and the mean for the first pecks was .43 (*SD* = *.*32).

Terminal-link responding on the suboptimal alternative showed consistent evidence that the terminal-link signals were discriminable, with the relative rate of responding on the signal for food exceeding .90 for all birds in both conditions.

## Experiment 2

In Experiment 2, all pigeons started in a baseline condition with an intermediate initial-link duration and then were split into two groups for the second condition, one with a shorter choice phase and the other with a longer duration. The initial- and terminal-link values were selected so the SiGN model predicted symmetrical shifts from approximate indifference in the baseline condition to preference for the suboptimal alternative for one group and preference for the optimal alternative for the other group.

### Method

#### Subjects

The subjects consisted of ten pigeons, eight of which also participated in Experiment 1.

Birds 6125 and 392 replaced Bird 822 (the sick bird removed from Experiment 1) and Bird 6 (who was deceased). The pigeons were housed and maintained as described in Experiment 1.

#### Apparatus

The experimental chambers and equipment described in Experiment 1 were also used in Experiment 2.

#### Procedure

##### Pretraining

Prior to beginning the experiment, each bird received the same pretraining experience described in Experiment 1.

##### Training

The general procedure was as described in Experiment 1 and shown in Fig. 1, but with the following changes to the stimuli and reinforcement schedules. A black ✕ and yellow and red keylights were presented on the left response key, and a black ✕ and blue and white keylights were presented on the right response key. The probability was .2 for entry to both the yellow and blue terminal links and .8 for entry to both the red and white terminal links. In the first condition, the initial-link schedule was the same for all 10 birds and consisted of a single VI 4.75-s timer and the terminal-link was a fixed-time 8-s schedule. This phase continued for 17 sessions, after which the birds were randomly divided into two groups of five. For one group, the initial link was shortened to a VI 1.7-s schedule and for the other it was lengthened to a VI 35-s schedule. The black ✕ initial-link stimuli were replaced with squares in the second phase, which continued for an additional 27 sessions.

### Results

**Fig. 3:**
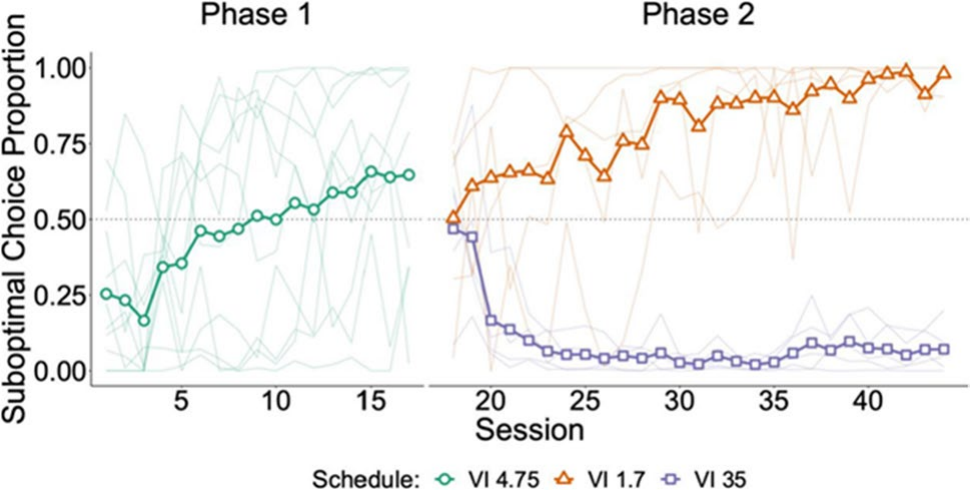
Experiment 2 results. *Note.* Suboptimal choice proportion across sessions. Bold lines represent mean choice proportion and thin lines represent individual subject data. Schedule is denoted by both shape and color. VI is the variable-interval schedule (in s) in the initial link. (Color figure online)

Choice proportion calculations again used the last three sessions of each condition and Fig. 3 shows the development of preference. When the choice schedule was VI 4.75, choices favored the suboptimal alternative (*M* = *.*65, *SD* = *.*30) with considerable variability across subjects. This variability is unsurprising given that this schedule was chosen to approximate indifference, allowing idiosyncratic preferences to emerge.

In the second phase, subjects switched to the VI 1.7-s schedule all strongly preferred the suboptimal alternative (*M* = *.*96, *SD* = *.*03), and subjects switched to the VI 35-s schedule all strongly preferred the optimal alternative (*M* = *.*06, *SD* = *.*06). Thus, once the value of the two alternatives diverged, variability decreased sharply.

Two paired *t* tests and one Welch independent *t* test were conducted to compare pairwise combinations of schedule types (Welch, 1938). The *p* values were adjusted for multiple comparisons using the method by Holm (1979), and Hedges’s *g* effect sizes with corresponding 95% confidence intervals (Borenstein et al., 2009). Within-subject comparison of the VI 4.75 and VI 1.7-s schedules indicated a significant difference; *t*(4) = *−*3*.*10, *p* = 0*.*036, *g* = *−*1*.*24, 95% CI [*−*2*.*47*, −*0*.*01]. Comparison between the VI 4.75 and VI 35-s schedules also showed a significant difference; *t*(4) = 4*.*27, *p* = 0*.*026, *g* = 1*.*39, 95% CI [0*.*34*,* 2*.*45]. Between-subject comparison of the VI 1.7-s and 35-s schedules was also statistically significant with a large effect; *t*(5*.*36) = 29*.*41, *p <* 0*.*001, *g* = 16*.*8, 95% CI [8*.*87*,* 24*.*73]. First peck data showed the same pattern of initial-link effects (see Appendix B for data and analysis). As in Experiment 1, the relative response rate on the terminal-link signal for food on the suboptimal alternative exceeded .90 for all birds in all conditions.

## General discussion

Both experiments show clear effects of temporal context on suboptimal choice, demonstrating that choice of the suboptimal alternative reliably decreases with increases in initial-link duration. These results, together with evidence of an effect of terminal-link duration (McDevitt et al., 2018; Spetch et al., 1990, 1994) confirm that temporal variables critically determine suboptimal preference. These changes in preference are unlikely to be due to artifacts in the arrangement of the VI schedules because the single timer eliminated inadvertent reinforcement for switching between alternatives and reduced the discrepancy between programmed and obtained schedule values.

The first peck measurement demonstrates that the effect of initial-link duration is evident on the first pecks, not just overall choice, and this effect is consistent for every subject. This supports the notion that the relative value of the two alternatives is altered by changes in temporal context and is not due to the dynamics associated with additional time available for responding.

An examination of the first peck data in the longest initial-link conditions (VI 30 and VI 35) is particularly interesting, as these conditions provided the greatest opportunity for preference to shift after the initial response. These data (Table 1) show that some subjects’ choices became more optimal after the first peck. Moreover, the three subjects tested in both conditions were consistent in the degree to which their first peck proportions compared to the overall choice proportions. The first response may be more sensitive to the conditioned reinforcement that underlies suboptimal choice. Laude et al. (2014) reported that individual differences in suboptimal choice were correlated with a measure of “impulsivity” derived from a hyperbolic-delay discounting procedure. Further research might explore whether individual differences in first pecks with long initial links correlate with measures of “impulsivity.”

**Table 1:** Suboptimal choice proportions for conditions with long VI durations

	Exp. 1 VI 30		Exp. 2 VI 35	
Bird	First Peck VI 30	Overall VI 30	First Peck VI 35	Overall VI 35
34	0.00	0.01	0.00	0.00
43	0.43	0.58		
48	0.77	0.22	0.30	0.14
1332	0.27	0.05		
1349	0.70	0.13	0.40	0.03
4			0.00	0.03
392			0.20	0.12
Mean	0.43	0.20	0.18	0.06

The striking effect of initial-link duration on suboptimal choice shown here is consistent with the role of temporal variables in associative learning and may reflect a more general phenomenon in which the value of a signal for reward depends on the temporal context in which it is embedded. These results are consistent with the findings of other procedures which found effects of temporal context on preference for information (Alsop & Davison, 1986; Hursh & Fantino, 1974). Further research might systematically explore how temporal context affects other forms of non-instrumental information seeking, a topic that has generated considerable recent interest within neuroscience and human decision making (Bromberg-Martin & Monosov, 2020; FitzGibbon et al., 2020; Liew, Embrey, & Newell, 2023b; Rodriguez Cabrero et al., 2019)

**Fig. 4:**
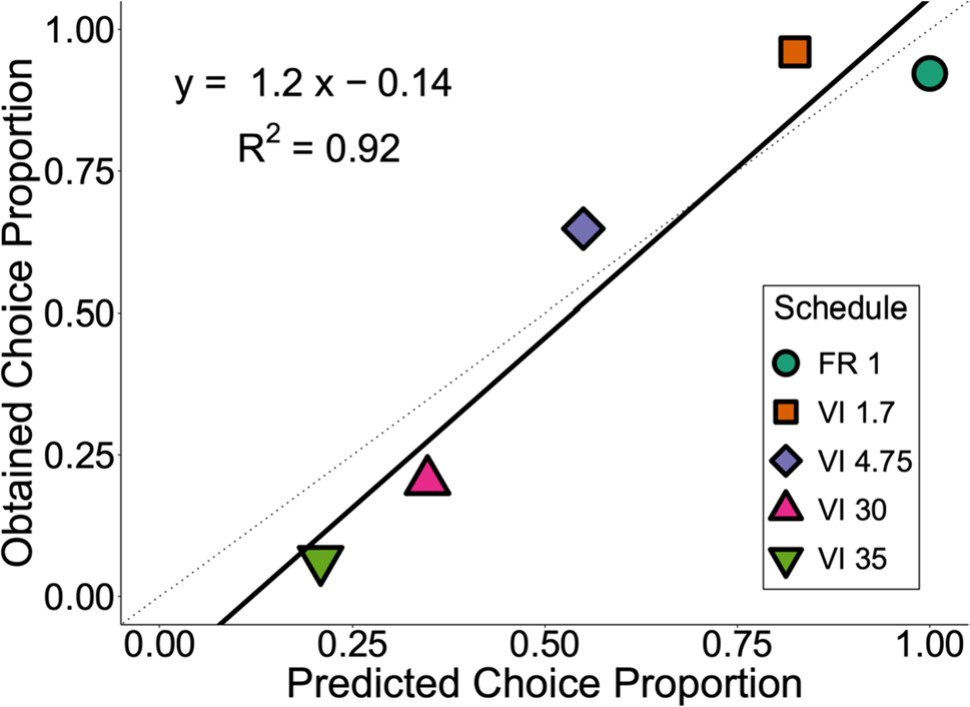
Obtained choice proportions as a function of predicted proportions*. Note.* The solid black line represents the fit of an ordinary least-squares regression line. The thin dotted line represents the hypothetical 1:1 correspondence (i.e., a perfect fit) of predicted to obtained values. FR 1 indicates a fixed-ratio 1 schedule and VI indicates a variable-interval schedule (in s) in the initial link. (Color figure online)

The inverse relationship between initial-link duration and suboptimal choice observed here supported the a priori quantitative predictions of the SiGN model (Dunn et al., 2024). According to this model, both the relative conditioned reinforcement and the relative rate of primary reinforcement shift toward the optimal alternative with longer initial links. Figure 4 shows the SiGN model’s predicted values against obtained choice proportions from both experiments. The correlation between predicted and observed values was very strong, *r* = .96, *t*(3) = 5.80, *p* = .010, and the intercept (*b*_0_) and slope (*b*_1_) did not significantly differ from 0 and 1, respectively, *b*_0_ = −0.14, *t*(3) = −1.05, *p* = .370; *b*_1_ = 1.20, *t*(3) = 0.96, *p* = .408.

The SiGN model predicts choice by considering the combined impact of primary and conditioned reinforcement. However, a different approach to explaining choice that also considers temporal factors is the information-theoretic model (Cunningham & Shahan, 2018). From that perspective, suboptimal choice arises when (1) the suboptimal terminal-link stimuli convey more information about when food will occur, and (2) the delay to terminal-link stimuli is much smaller than the delay to food when a choice is made, thereby biasing animals to base decisions on the temporal informativeness of terminal-link stimuli rather than food rate. This model predicts an increase in optimal choice with increases in initial-link duration by altering the competition between temporal information that favors the suboptimal alternative and relative rate of food that favors the optimal alternative. By adjusting the parameter that modulates bias for using information to make decisions (m) and sensitivity to primary reinforcement (b), the information-theoretic model can provide a good fit to the data presented here (P. Cunningham, personal communication, October 9, 2023). However, doing so requires parameter values that differ considerably from those obtained in Cunningham and Shahan’s (2018) analysis of suboptimal choice data. This highlights a limitation of models with free parameters—namely, a challenge in offering decisive a priori predictions. Nevertheless, the information-theoretic model provides a versatile account of choice in concurrent-chains procedures that corresponds nicely with a cognitive framework valuing information as an explanatory variable.

With the exception of the information-theoretic model, other models of suboptimal choice do not address initial-link duration, which is surprising given that temporal context is a central concept in understanding behavior (e.g., Balsam et al., 2010; Ludvig et al., 2012; Molet & Miller, 2014). Our experiments highlight the need to incorporate temporal variables in models of suboptimal choice. Uncovering mechanisms underlying suboptimal choice in animals adds to our understanding of how organisms respond to reward uncertainty, which may have implications for human behaviors such as risky choice and gambling (e.g., Matthews et al., 2023; Zentall, 2023).

In a clear parallel to suboptimal choice in pigeons, humans and other animals seek advance information about uncertain rewards, sometimes foregoing rewards, paying money, or enduring pain to obtain information even when it does not affect the outcome (e.g., Bennett et al., 2016; Blanchard et al., 2015; Bode et al., 2023; Rodriguez Cabrero et al., 2019). Chasing of prediction error has been proposed as a mechanism of suboptimal choice (Zhu et al., 2017), and the neural circuitry underlying prediction errors and information seeking has been a focus of considerable research (e.g., Bromberg-Martin et al., 2024; Brydevall et al., 2018). Delays between choice and reward can increase preference for information in humans (e.g., Iigaya et al., 2020) suggesting that temporal factors are important. However, the importance of choice duration has been overlooked in the information-seeking literature. Our study shows that altering the choice duration has a dramatic effect and changes which alternative pigeons prefer. This demonstration of a critical role for choice period duration suggests that temporal context warrants further exploration in understanding information seeking.

## Supplementary Information

Below is the link to the electronic supplementary material.

Supplementary file1 (PDF 190 KB)
