# Supplementary material for: Temporal context effects on suboptimal choice
Source: Psychon Bull Rev. 2024 May 17;31(6):2737–45. doi: 10.3758/s13423-024-02519-y (PMC11680616; doi:10.3758/s13423-024-02519-y)
Supplement: Supplementary file 1 — Supplementary file1 (PDF 190 KB) [file 13423_2024_2519_MOESM1_ESM.pdf]

Appendix A

Application of the SiGN Model to the FR 1 and VI 30 conditions in Experiment 1

The SiGN model predicts specific choice proportions based only on the temporal and probability variables embedded in the suboptimal choice procedure. It does not employ free parameters. Predictions can be generated quickly at https://jpisklak.shinyapps.io/SiGN_Calc/. Here we work through the calculations required for choice between signaled 20% and unsignaled 50% with the two initial-link schedules used in Experiment 1. To enhance readability, calculations have been rounded, resulting in a minor degree of discrepancy. The purpose of each step is described in detail in Dunn et al. (2024).

The SiGN model is summarized in the equation:

$$\frac{R_a}{R_a + R_b} = \frac{r_a \delta_a}{r_a \delta_a + r_b \delta_b} \quad \text{when } \delta_a > 0, \delta_b > 0$$

$$= 1 \quad \text{when } \delta_a > 0, \delta_b < 0$$

$$= 0 \quad \text{when } \delta_a < 0, \delta_b > 0$$

where R_a and R_b denote the choice responses, r_a and r_b stand for the rates of unconditioned reinforcement, and δ_a and δ_b represent the conditioned reinforcement on alternatives A and B, respectively.

Steps in the calculation for the FR 1 condition

1. Determine rate of unconditioned reinforcement (food) separately for alternatives A and B (r_a and r_b).

   (a) Determine the average time in the initial link per food delivery. This includes time spent leading to the unreinforced terminal links. Assuming 1 s to make a peck:

   Alternative A (20% food) $= 1 \div 0.2 = 5$ s

   Alternative B (50% food) $= 1 \div 0.5 = 2$ s

   (b) Determine the average time spent in the terminal links per food delivery. On alternative A, there are five terminal links per reinforcer. Four of these signal no food

and one signals the delivery of food. Terminal links signaling no food should only have the first 1 s of their duration considered (see Dunn et al., 2024), while full and partial signals for food should have the full 20 s applied. On alternative B, there are two unsignaled terminal links per food delivery. Thus:

Alternative A: $1+1+1+1+20 = 24$ s

Alternative B: $20+20 = 40$ s

(c) Sum the results of steps (a) and (b).

Alternative A: $5+24 = 29$ s

Alternative B: $2+40 = 42$ s

(d) The inverse of each value is the rate, that is, the average number of food deliveries that could be obtained per second:

Alternative A: $r_a = 1 \div 29 = 0.0345$

Alternative B: $r_b = 1 \div 42 = 0.0238$

2. Determine conditioned reinforcement effects.

(a) Determine overall time to food, T, by multiplying the inverse of r_a and r_b by the relative probability of reinforcement on alternative A and B respectively:

$$T = \left(\frac{1}{0.0345}\right)\left(\frac{0.2}{0.2+0.5}\right) + \left(\frac{1}{0.0238}\right)\left(\frac{0.5}{0.2+0.5}\right)$$
$$= 38.29$$

(b) When an alternative is chosen, what is the average time to food at the point of transition to that terminal link? This will include time spent recycling through the initial links when terminal links do not end in food. Remember each signaled period of non-reinforcement is equivalent to 1 s.

On alternative A, the time to food after choice is 20 s on 20% of the terminal-link entries and the other 80% of the time, it is 1 s plus T (see step 2a) so the times to food are:

For Review Only

Alternative A: $(20 \times 0.2) + ((1 + 38.29) \times 0.8) = 35.43$ s

Alternative B: $(20 \times 0.5) + ((20 + 38.29) \times 0.5) = 39.14$ s

(c) Determine average delay reduction on each alternative by subtracting the times spent in terminal links as calculated in the previous step from T

Alternative A: $38.29 - 35.43 = 2.86$ s

Alternative B: $38.29 - 39.15 = -0.85$ s

(d) Determine Good News Bonus. When a terminal-link stimulus is presented, is the time to food less than the average time on that alternative (from Step 2b)? If so, then the additional delay reduction is the good news bonus. In this case only alternative A signals a bonus delay reduction. Thus,

Alternative A: $35.43 - 20 = 15.43$ s

Alternative B: Has no good news bonus.

Calculate the balance of conditioned and unconditioned reinforcement (β) on the suboptimal alternative. The good news bonus is then adjusted by β as determined by the durations of the initial link and the signal for food (S^+).

$$\beta = \log_{10}\left(1 + \frac{S^+ \text{ terminal-link duration}}{\text{initial-link duration}}\right)$$

$$= \log_{10}\left(1 + \frac{20}{1}\right)$$

$$= 1.32$$

The good news bonus is the additional delay reduction multiplied by β. The good news bonus is

Alternative A: $15.43 \times 1.32 = 20.37$

Alternative B: There is no good news bonus.

(e) Determine total conditioned reinforcement for each alternative, δ_a, δ_b, by adding the good news bonus (if any) to the average delay reduction for each alternative.

For Review Only

Alternative A: $\delta_a = 2.86 + 20.37 = 23.23$ s

Alternative B: $\delta_b = -0.85$ s

Since $\delta_a > 0$ and $\delta_b < 0$, the predicted choice proportion will just be equal to 1 in favor of the suboptimal alternative; nevertheless, we proceed with the calculations to illustrate the full process for circumstances in which $\delta_a > 0$ and $\delta_b > 0$

3. Calculate the choice proportion:

$$\frac{R_a}{R_a + R_b} = \frac{r_a \delta_a}{r_a \delta_a + r_b \delta_b}$$

$$= \frac{0.0345 \times 23.23}{(0.0345 \times 23.23) + (0.0238 \times -0.85)}$$

$$= 1.03$$

Steps in the calculation for the VI 30 condition

More detail on each step is provided in the example above. Note that the following applies when there is a single initial-link timer as used in the present study. The calculation in Step 2a differs for initial links with concurrent VIs or unequal VIs (for details see Dunn et al., 2024).

1. Determine rate of food deliveries separately for alternatives A and B (r_a and r_b).

   (a) The average times in the initial link per food delivery are:

   Alternative A: (20% food) $= 30 \div 0.2 = 150$ s

   Alternative B: (50% food) $= 30 \div 0.5 = 60$ s

   (b) The average times spent in the terminal links per food delivery are:

   Alternative A: $1 + 1 + 1 + 1 + 20 = 24$ s

   Alternative B: $20 + 20 = 40$ s

   (c) Sum the results of steps (a) and (b).

   Alternative A: $150 + 24 = 174$ s

For Review Only

Alternative B: $60+40=100$ s

(d) Food deliveries per second are:

Alternative A: $r_a = 1 \div 174 = 0.005747$

Alternative B: $r_b = 1 \div 100 = 0.01$

2. Determine conditioned reinforcement effects.

(a) The overall time to food, T, is

$$T = \left(\frac{1}{0.005747}\right)\left(\frac{0.2}{0.2+0.5}\right) + \left(\frac{1}{0.01}\right)\left(\frac{0.5}{0.2+0.5}\right)$$
$$= 121.14$$

(b) The average times to food at the point of transition to a terminal link are:

Alternative A: $(20\times 0.2)+((1+121.14)\times 0.8) = 101.71$ s

Alternative B: $(20\times 0.5)+((20+121.14)\times 0.5) = 80.57$ s

(c) The average delay reductions for a terminal-link entry are:

Alternative A: $121.14 - 101.71 = 19.43$ s

Alternative B: $121.14 - 80.57 = 40.57$ s

(d) The Good News Bonus:

Alternative A: The additional delay reduction is $101.71 - 20 = 81.71$ s

Alternative B: There is no additional delay reduction.

$$\beta = \log_{10}\left(1+\frac{20}{30}\right) = 0.22$$

Alternative A: The good news bonus is $81.71 \times 0.22 = 17.98$

Alternative B: There is no good news bonus.

(e) The total conditioned reinforcement for each alternative is:

Alternative A: 19.43 + 17.98 = 37.41 s

Alternative B: 40.57 s

For Review Only

3. Calculate the choice proportion:

$$\frac{R_a}{R_a + R_b} = \frac{0.005747 \times 37.41}{(0.005747 \times 37.41) + (0.01 \times 40.57)}$$
$$= 0.35$$

For Review Only

Appendix B

Supplementary Analysis and First Peck Results

The individual subject first peck choice proportions (calculated for the suboptimal alternative and averaged over the last three sessions in each condition) are shown below in Table B1 and Table B2, for Experiments 1 and 2, respectively. The option chosen on the first peck was added as a measure at the end of the second phase of Experiment 1, so data are only available for birds that received the VI 30 condition in phase 2.

Experiment 1

Each of the five birds that had the VI 30 condition in phase 2 showed first peck choice proportions with the VI 30 ($M = .43$, $SD = .32$) that were lower than those observed in the FR 1 condition ($M = .95$, $SD = .04$). The first choice proportions for these conditions were significantly different ($t(4) = 3.99$, $p = .016$, $g = 1.16$, 95% CI $[0.29, 2.03]$).

Experiment 2

Experiment 2. As with overall choice proportions, the mean proportion of first pecks in the initial VI 4.75-s schedule condition slightly favored the suboptimal alternative ($M = .62, SD = .31$). In phase 2, preference diverged when birds switched to VI 1.7-s and VI 35-s schedules. The VI 1.7-s schedule produced a notably strong suboptimal preference ($M = .93$, $SD = .06$), whereas the VI 35-s schedule resulted in a comparatively optimal preference ($M = .18$, $SD = .18$). Collectively, the pattern of first peck results mirrors those observed in the analysis of the pigeons' total responses. These results were analyzed in a manner similar to the overall choice proportions reported in Experiment 2. The within-subject comparison between the VI 4.75 and VI 1.7-s schedules yielded a statistically significant difference ($t(4) = -3.93$, $p = .033$, $g = -1.29$, 95% CI $[-2.32, -0.27]$). Similarly, the comparison between the VI 4.75 and VI 35-s schedules was also significantly different ($t(4) = 3.98$, $p = .033$, $g = 1.47$, 95% CI $[0.24, 2.70]$). The between-subject comparison of the VI 1.7 and VI 35-s schedules exhibited a statistically significant difference with a pronounced effect size ($t(4.96) = 8.89$, $p = .001$, $g = 5.08$, 95% CI $[2.37, 7.79]$).

For Review Only

Table B1

Experiment 1 First Peck Choice Proportions (CP) to the Suboptimal Alternative

	Initial-Link Schedule	
Bird	FR 1	VI 30
34	0.90	0.00
43	0.90	0.43
48	0.97	0.77
1332	0.97	0.27
1349	1.00	0.70
Mean	0.95	0.43

For Review Only

Table B2

Experiment 2 First Peck Choice Proportions (CP) to the Suboptimal Alternative

	Initial-Link Schedule		
Bird	VI 4.75	VI 35	VI 1.7
4	0.93	0.00	
34	0.13	0.00	
48	0.70	0.30	
392	0.93	0.20	
1349	0.93	0.40	
42	0.23		0.87
43	0.93		0.97
1311	0.43		0.87
1332	0.37		0.97
6125	0.60		1.00
Mean	0.62	0.18	0.93

For Review Only
